# The Relation Between Hemiparetic Gait Patterns and Walking Function After Stroke, as Measured with Wearable Sensors

**DOI:** 10.1007/s10439-025-03754-7

**Published:** 2025-05-14

**Authors:** Brice Thomas Cleland, Madeline Kim, Sangeetha Madhavan

**Affiliations:** Brain Plasticity Lab, Department of Physical Therapy, College of Applied Health Sciences, University of Illinois Chicago, 1919 W. Taylor St., Chicago, IL 60612 USA

**Keywords:** Stroke, Stroke rehabilitation, Locomotion, Lower extremity, Lower limb function, Gait

## Abstract

**Purpose:**

After stroke, walking is characterized by hemiparetic patterns, quantified with force sensitive walkways and motion capture systems. Some joint-level kinematic patterns of walking also can be obtained with wearable sensors. The purpose of this project was to measure joint-level kinematic patterns during walking with wearable sensors and determine the association with walking speed and endurance in individuals with chronic stroke.

**Methods:**

In this cross-sectional observational study, participants donned APDM Opal wearable sensors during walking tests (10-meter walk test or 6-min walk test). We extracted joint-level kinematic variables of elevation at midswing, circumduction, foot strike angle, and toe-off angle. Associations of each variable with walking speed and endurance were tested, and significantly associated variables were entered into a regression model.

**Results:**

68 individuals with chronic stroke were included. We found that the less affected foot strike angle, less affected toe-off angle, and more affected toe-off angle were significant predictors of walking speed (*R*^2^ ≥ 0.71, *p* < 0.001). Less affected toe-off angle, more affected foot strike angle, and more affected toe-off angle were significant predictors of walking endurance (*R*^2^ ≥ 0.67, *p* < 0.001).

**Conclusion:**

We found consistent evidence that greater toe-off angle (may reflect greater push-off) and lesser foot strike angle (may reflect lesser foot drop) were important predictors of greater walking speed and endurance. Our results suggest that wearable sensors can provide important information about joint-level kinematic patterns that are important for walking function. This information could help therapists target interventions toward specific deficits or compensatory patterns to improve walking.

## Introduction

After stroke, walking is characterized by hemiparetic patterns. There are extensive observations of asymmetrical spatial (e.g., step length) and temporal (e.g., stance time) patterns during walking after stroke [1–9]. The more affected limb produces more braking and less propulsive work than the less affected limb, and accounts for ~ 40% of the total work throughout the walking cycle [10–13]. Accordingly, the more affected limb has lower kinetic energy and decreased joint moments than the less affected limb [3, 14, 15]. Joint-level kinematic changes include stroke-related deficits such as (1) stiff-knee gait, (2) foot drop, and (3) reduced push-off, and subsequent compensatory patterns including (4) hip/pelvic hiking, (5) circumduction, and (6) stepping gait [1, 3, 16–19].

These walking patterns often develop during acute stroke (when motor deficits are the greatest) and some are reinforced in stroke rehabilitation to help quickly improve function [20, 21]. However, the persistence of these movement patterns into the chronic phase of stroke may have negative effects on walking function. Some studies have found that increased spatiotemporal asymmetry is associated with decreased walking speed [2, 4, 6, 7, 9, 22–24]. Decreased ankle plantarflexion strength in the more affected limb and increased force/power production asymmetry between limbs are associated with decreased walking speed [6, 10, 25–27]. Conversely, greater ankle and hip power generation are associated with faster walking speed [18]. Some work has shown that slower walking speed is associated with stiff-knee gait, foot drop, reduced push-off, hip/pelvic hiking and tilt, and vaulting [3, 11, 18, 19, 28–31]. In contrast, greater circumduction is associated with faster walking speeds [18, 30].

From a clinical perspective, there are some limitations to obtaining and using walking spatiotemporal kinematic, kinetic, and/or joint-level kinematic measures to assess hemiparetic gait patterns after stroke. Spatiotemporal kinematic measurements provide broad information about walking characteristics and symmetry, but these outcomes may be more difficult to target with walking interventions than kinetic or joint-level kinematic measures. Walking kinetics provide important data, but measuring these outcomes requires force sensitive walkways/force plates. Similarly, almost all prior work in joint-level kinematics of walking has used motion capture systems. Force sensitive walkways/force plates and motion capture systems have some notable limitations for clinical use: they are expensive, they use a large and fixed amount of space, they require specialized expertise to obtain usable outcomes, and they often require extensive setup time [32].

With recent developments in the fields of wearable sensors and cameras, information about joint-level kinematic patterns of walking can now be obtained without the same cost and space requirements as traditional force measurement and motion capture systems. Many sensor-based systems, including APDM Opal, MTw Awinda, Fox HikoB, Physilog, Axivity AX3, XSens, and Baiobit, have shown good agreement with gold-standard force measurement and motion capture systems [33–41]. However, almost all these systems lack a clinician-friendly interface and require specialized expertise to obtain useable metrics of walking. Other limitations include the use of a single sensor, difficulties with ankle movement detection, and a restricted number of outcome measures. Camera or smartphone-based systems (e.g., OpenCap, SMARTGAIT, Azure Kinect, Odontate) are growing in popularity, but many of these systems have estimation errors, particularly in individuals with pathological gait and of the ankle, and tend to lack a clinician-friendly interface [42–48].

The purpose of this project was to measure joint-level kinematic patterns during walking with wearable sensors and determine the association with walking speed and endurance in individuals with chronic stroke. Participants donned wearable sensors during walking tests, and we extracted joint-level kinematic variables that best represented some of the hemiparetic gait patterns highlighted above. We hypothesized that individuals with less pronounced hemiparetic gait patterns (both stroke-related deficits and compensatory patterns), as measured with the wearable sensors, would have faster walking speeds.

In this study, we chose to use APDM Opal inertial measurement units (IMUs) and the corresponding Mobility Lab software because this system is among the most clinician-friendly sensor systems available. The system uses multiple, easy-to-apply sensors and automatically outputs joint-level kinematic measurements that are not included in other software but provide important insights into walking after stroke. The selected joint-level kinematic variables could be obtained easily by clinicians without any specialized analytic knowledge. We are not aware of any previous work that has measured joint-level kinematics with wearable sensors and examined the relation with walking function.

## Materials and Methods

### Participants

Data for the current study were used from screening and pre-test sessions for a clinical trial (ClinicalTrials.gov: NCT04477330, registration date: 07/20/2020). Data were collected from 04/2021 to 04/2023. For this overarching study, participants were included if they were > 18 years of age, had a monohemispheric stroke > 6 months before enrollment, and had residual hemiparetic gait deficits. Individuals were excluded if they had uncontrolled cardiorespiratory or metabolic diseases, cognitive impairment, or lesions of the brainstem or cerebellum. This study was approved by the institutional review board at the University of Illinois Chicago and conformed to the ethical standards in the Declaration of Helsinki. All participants provided written, informed consent. Data for this study are openly available at: 10.25417/uic.25360474. The STROBE reporting guidelines were used in this manuscript.

### Walking and Clinical Tests

Participants completed 2-3 trials of the 10-meter walk test (10 mWT) at both comfortable and maximal speeds. Time to walk the middle 6 meters was recorded with a stopwatch. Walking time was averaged across trials and average walking speed was calculated (6 meters/average walking time). Participants who qualified for the clinical trial performed the 6-min walk test (6 mWT). Participants walked back and forth down a 30-meter hallway for 6 min, and the distance covered by the end of the test was recorded. For all walking tests, participants wore their normal footwear.

### Sensors & System

**Fig. 1:**
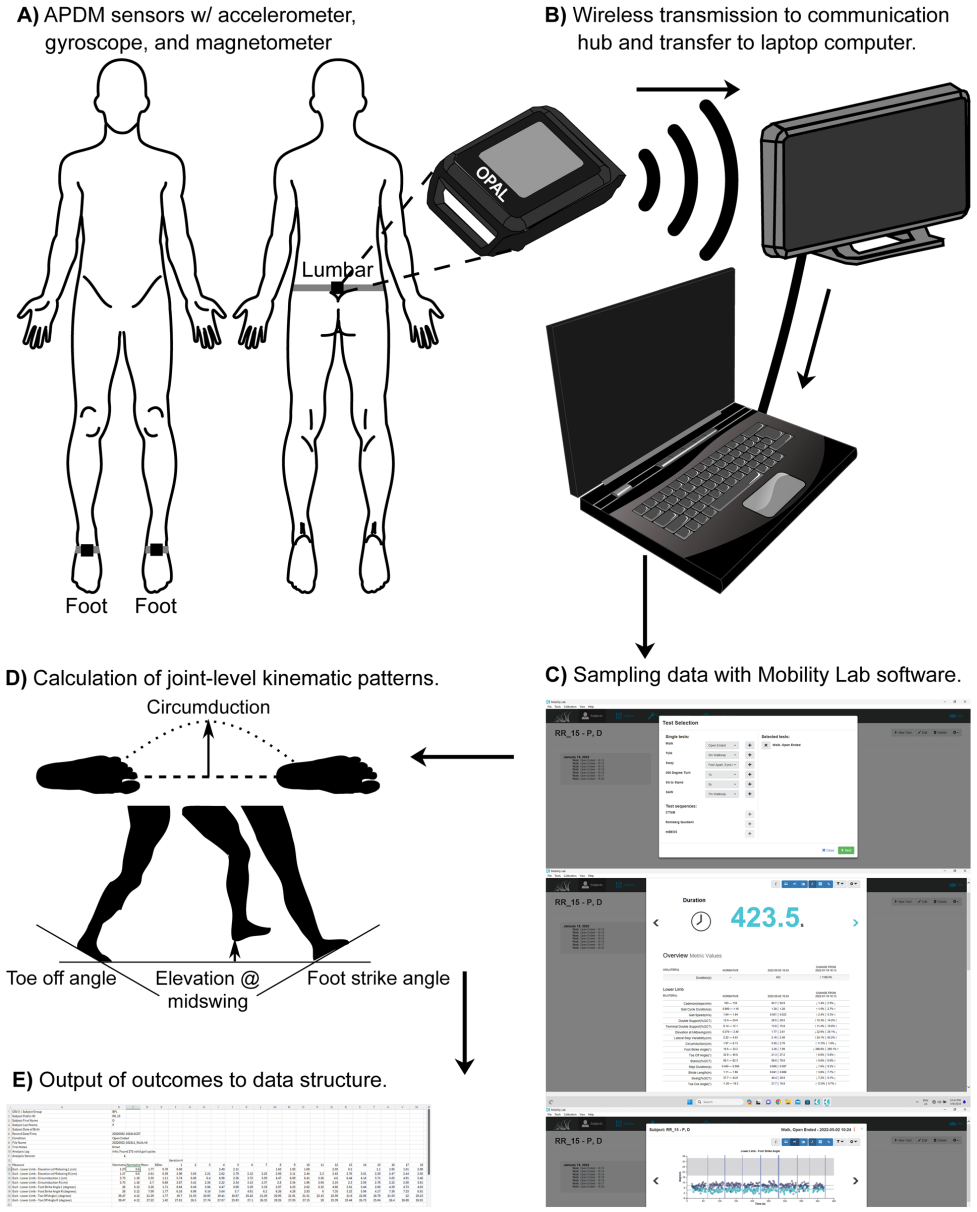
Experimental setup and data flow. **A** Participants wore APDM Opal sensors (50 × 40 × 14 mm) strapped around each foot and on the lumbar region. Foot sensors were placed over the shoe, centered on the top of the foot, with an approximate location over the base of the metatarsals. Lumbar sensors were centered on the low back at the base of the spine (immediately superior to the L5 vertebra). The sensors contain a three-axis accelerometer (± 200 g), a three-axis gyroscope (± 2000°/s), a three-axis magnetometer (± 8 Gauss), and a barometer (300–1100 hPa). **B** Data were sampled at 20–128 Hz, wirelessly transmitted to a wireless communication hub, and transferred to a computer. **C** Data were sampled with Mobility Lab software. **D** Key outcome metrics (elevation at midswing, circumduction, foot strike angle, and toe-off angle were calculated. **E** Outcome variables were exported to a data structure. Lines with arrows show direction of data flow

During all walking tests, participants wore wireless sensors (Opal V2R, ADPM Wearable Technologies Inc., Portland, OR, USA) over both feet and the lumbar spine. Foot sensors were placed over the shoe, centered on the top of the foot, with an approximate location over the base of the metatarsals. Lumbar sensors were centered on the low back, at the base of the spine (immediately superior to the L5 vertebra). Opal sensors are small sensors (50 × 40 × 14 mm) containing a three-axis accelerometer (± 200 g), a three-axis gyroscope (±2000°/s), a three-axis magnetometer (± 8 Gauss), and a barometer (300–1100 hPa). Data were sampled at 20–128 Hz, wirelessly transmitted to a wireless communication hub, transferred to a computer, and sampled with Mobility Lab software version 2 [49]. Fig. 1 provides a visual representation of this experimental setup.

Signals were collected for the duration of each walking trial (< 90 sec for the 10 mWT and 360 sec for the 6 mWT). Raw data output from the devices were calculated as described in the APDM Technical Guide (https://share.apdm.com/documentation/TechnicalGuide.pdf). Data from all sensors were synchronized within the Mobility Lab software by interpolating (bandlimited windowed sinc interpolation) to the same sample rate. Mobility Lab software uses several open source software products (https://support.apdm.com/hc/en-us/articles/214505146-Open-Source-Software-OSS-used-in-APDM-software-products) and internal algorithms to calculate the joint-level kinematic patterns outlined below from raw sensor data. Algorithms used in the Mobility Lab software have been validated by comparing the Opal system output variables with the same variables from the GaitRite Mat and an optical motion capture system [36, 37].

### Joint-Level Kinematic Patterns

Four kinematic pattern variables were determined for every gait cycle by the Mobility Lab proprietary software (definitions below from APDM User Guide): elevation at midswing, circumduction, foot strike angle, and toe-off angle. These variables provide information directly relevant to major joint-level kinematic changes that occur after stroke and are automatically calculated and output from the Mobility Lab software. Hence, clinicians could access this clinically relevant information without needing technical analytic expertise.

1. Elevation at midswing—“the height of the foot sensor measured at midswing, relative to its start position while standing.” Units are cm, with more positive values representing greater elevation. This variable was extracted to provide insight into several joint-level kinematic patterns, including stiff-knee gait, hip/pelvic hiking, and stepping gait (#1, #4, and #6 in the Introduction).
2. Circumduction—“the amount that the foot travels perpendicular to forward movement while swinging forward during an individual stride.” Units are cm, with more positive values representing greater circumduction. This variable was extracted to reflect the compensatory joint-level kinematic pattern of circumduction (#5 in the Introduction).
3. Foot strike angle—“the angle of the foot at the point of initial contact. The pitch of the foot when flat is zero and positive when the heel contacts first.” Units are degrees, with more positive values representing greater dorsiflexion. This variable was extracted to reflect the stroke-related joint-level kinematic pattern of foot drop (#2 in the Introduction).
4. Toe off angle—“the angle of the foot as it leaves the floor at push-off. The pitch of the foot when flat is zero.” Units are degrees, with more positive values representing greater plantarflexion. This variable was extracted to reflect the stroke-related joint-level kinematic pattern of reduced push-off (#3 in the Introduction).

After extracting these variables, analyses were performed in Matlab (R2021b, Mathworks, Natick, MA, USA). For each variable, individual datapoints > 4 standard deviations (SD) outside the mean value were removed (typically 0 datapoints were removed). Afterward, the mean value was taken across all gait cycles for each variable. If data were available from the 6 mWT, this trial was used for analyses. For participants who did not complete the 6 mWT, data were taken from the 10 mWT at a comfortable speed, and trials were concatenated before outlier removal and determination of mean.

### Statistical Analysis

To test for differences between the more affected and less affected limb, we performed independent samples t-tests. To test the association between each joint-level kinematic pattern with walking speed and walking endurance, we performed Pearson correlations. Because we found that many of the patterns were associated with walking speeds and endurance, we subsequently performed a forward multiple linear regression for each of the three dependent variables (comfortable and maximal 10 mWT speed and 6 mWT distance). In this forward linear regressions, all kinematic pattern variables with significant correlations with the outcome of interest were provided as potential predictors. Variables were entered sequentially into the models, ordered based on the magnitude of the partial correlation. New variables were added if their F-value had a probability of < 0.05 until this criterion was no longer met. Statistical analyses were performed in SPSS Statistics 25 (IBM, Armonk, NY, USA), with two-sided statistical testing with *α* = 0.05.

## Results

### Participants

Data from 68 individuals with chronic stroke are included in the results. Demographic information about participants, including age, sex, race/ethnicity, time since stroke, stroke type, more affected limb, and assistive device used during walking tests are included in Table 1.

**Table 1:** Demographics

*N* = 68	Mean (SD)	Range
Age	62 (10)	31–84
Height (m)	1.72 (0.11)	1.49–1.92
Gender (male/female)	44/24	
Race/ethnicity		
Black	40	
White, Not Hispanic or Latino	17	
White, Hispanic or Latino	7	
Asian	3	
More than one race	1	
Time since stroke (years)	8 (6)	1 – 26
Stroke type (ischemic/hemorrhagic/unknown)	42/19/7	
Stroke location (cortical, subcortical, unknown)	21/22/25	
More affected limb (left/right)	36/32	
AFO (Yes/No)	24/44	
Assistive device? (Yes/No)	11/57	Cane—4 Quad cane—4 Walker—3

*AFO* ankle-foot orthosis

### Joint-Level Kinematic Patterns

The extracted kinematic pattern variables and walking variables are shown in Table 2. One outlier (> 4 SD outside the group mean) was removed for analysis for elevation at midswing, circumduction, and toe-off angle, and 10 mWT walking speeds. One additional datapoint was missing for elevation at midswing. 6 mWT endurance was not tested in 23 participants who did not qualify for the clinical trial, leaving an *n* = 45. When comparing the more affected and less affected limb, we found that the more affected limb had less elevation at midswing (mean difference = − 0.7 [95% CI − 1.1, − 0.4], *t*[130] = − 4.2, *p* < 0.001), more circumduction (mean difference = 2.5 [95% CI 1.6, 3.5], *t*[132] = 5.5, *p* < 0.001), a lesser foot strike angle (mean difference = − 3.6 [95% CI − 6.5, − 0.8], *t*[134] = − 2.6, *p* = 0.01), and a lesser toe-off angle (mean difference = − 9.8 [95% CI − 12.2, − 7.4], *t*[132] = − 8.1, *p* < 0.001).

**Table 2:** Kinematic pattern and walking variables

Kinematic pattern variable	Limb	Mean (SD)	Range
10 mWT comfortable speed (m/s) (*n* = 67)		0.69 (0.28)	0.14–1.17
10 mWT maximal speed (m/s) (*n* = 67)		0.97 (0.44)	0.15–1.85
6 mWT endurance (m) (*n* = 45)		260 (87)	61–403
Elevation at midswing (*n* = 66)	More affected	1.3 (0.9)	0.2–3.7
	Less affected	2.1 (1.2)	0.3–4.9
Circumduction (*n* = 67)	More affected	5.3 (3.5)	0.1–15.6
	Less affected	2.8 (1.5)	0.18–7.97
Foot strike angle (*n* = 68)	More affected	10.9 (7.8)	− 2.7–27.8
	Less affected	14.5 (8.8)	− 7.1–30.7
Toe off angle (*n* = 67)	More affected	20.1 (7.6)	6.8–32.4
	Less affected	29.8 (6.4)	12.3–40.1

*10 mWT* 10-meter walk test, *6 mWT* 6-min walk test, *ROM* range of motion

**Fig. 2:**
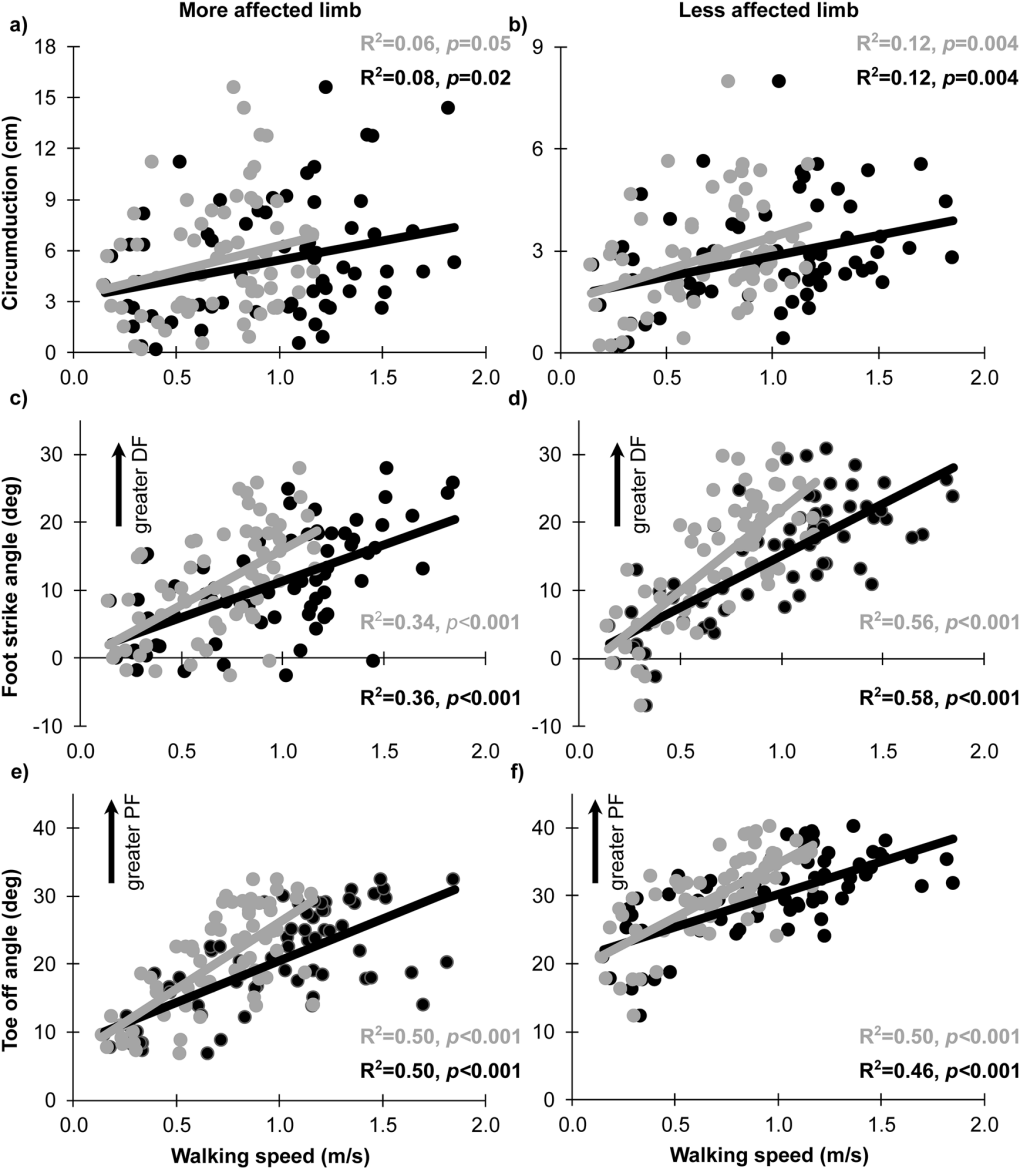
Correlations of select joint-level kinematic patterns with walking speed. Correlations are shown for comfortable (light gray) and maximal (black) walking speed with circumduction in the **a** more affected limb and **b** less affected limb; foot strike angle in the **c** more affected limb and **d** less affected limb; and toe-off angle in the **e** more affected limb and **f** less affected limb. Relations for the more affected limb are shown in the left column, while relations for the less affected limb are shown in the right column. Each dot represents data from a single participant. Solid lines are lines of best fit, and the associated *R*^2^ and *p*-values are shown. Note that for foot strike angle and toe-off angle, indicators are included to clarify what greater values represent (either more dorsiflexion [DF] or more plantarflexion [PF], respectively)

Fig. 2 shows correlations of circumduction (a, b), foot strike angle (c, d), and toe-off angle (e, f), in the more (left column) and less affected (right column) limb with comfortable (gray line) and maximal (black line) walking speed. We found significant correlations of faster comfortable walking speed with greater circumduction in the more affected (*R* = 0.24, *p* = 0.05) and less affected limb (*R* = 0.35, *p* = 0.004), greater foot strike angle in the more affected (*R* = 0.58, *p* < 0.001) and less affected limb (*R* = 0.75, *p* < 0.001), and greater toe-off angle in the more affected (*R* = 0.71, *p* < 0.001) and less affected limb (*R* = 0.71, *p* < 0.001). We found significant correlations of faster maximal walking speed with greater circumduction in the more affected (*R* = 0.29, *p* = 0.02) and less affected limb (*R* = 0.35, *p* = 0.004), greater foot strike angle in the more affected (*R* = 0.60, *p* < 0.001) and less affected limb (*R* = 0.76, *p* < 0.001), and greater toe-off angle in the more affected (*R* = 0.71, *p* < 0.001) and less affected limb (*R* = 0.68, *p* < 0.001).

**Fig 3:**
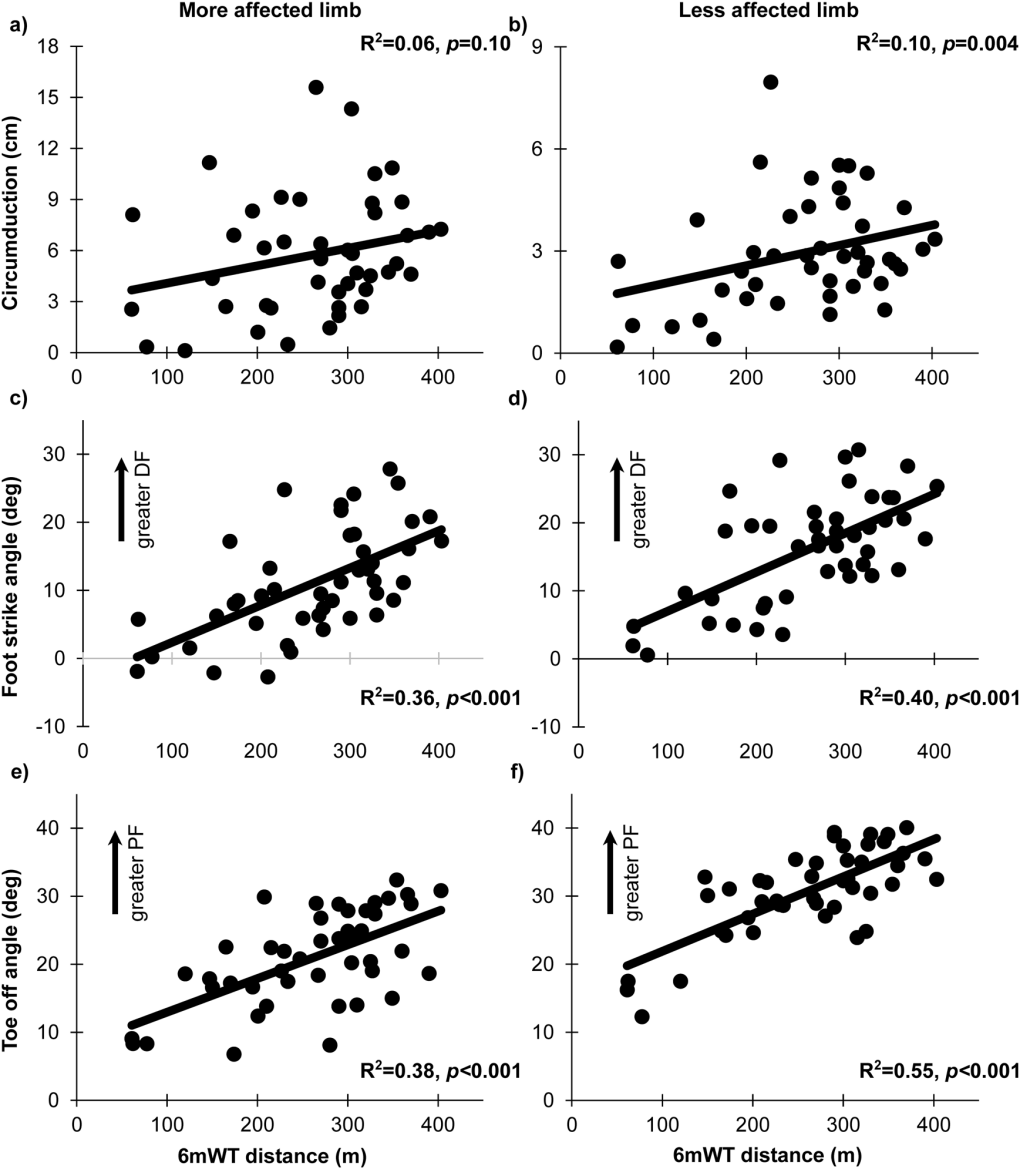
Correlations of select joint-level kinematic patterns with walking endurance. Correlations are shown for 6 mWT distance with circumduction in the **a** more affected limb and **b** less affected limb; foot strike angle in the **c** more affected limb and **d** less affected limb; and toe-off angle in the **e** more affected limb and **f** less affected limb. Relations for the more affected limb are shown in the left column, while relations for the less affected limb are shown in the right column. Each dot represents data from a single participant. Solid lines are lines of best fit, and the associated *R*^2^ and *p*-values are shown. Note that for foot strike angle and toe-off angle, indicators are included to clarify what greater values represent (either more dorsiflexion [DF] or more plantarflexion [PF], respectively)

**Fig 4:**
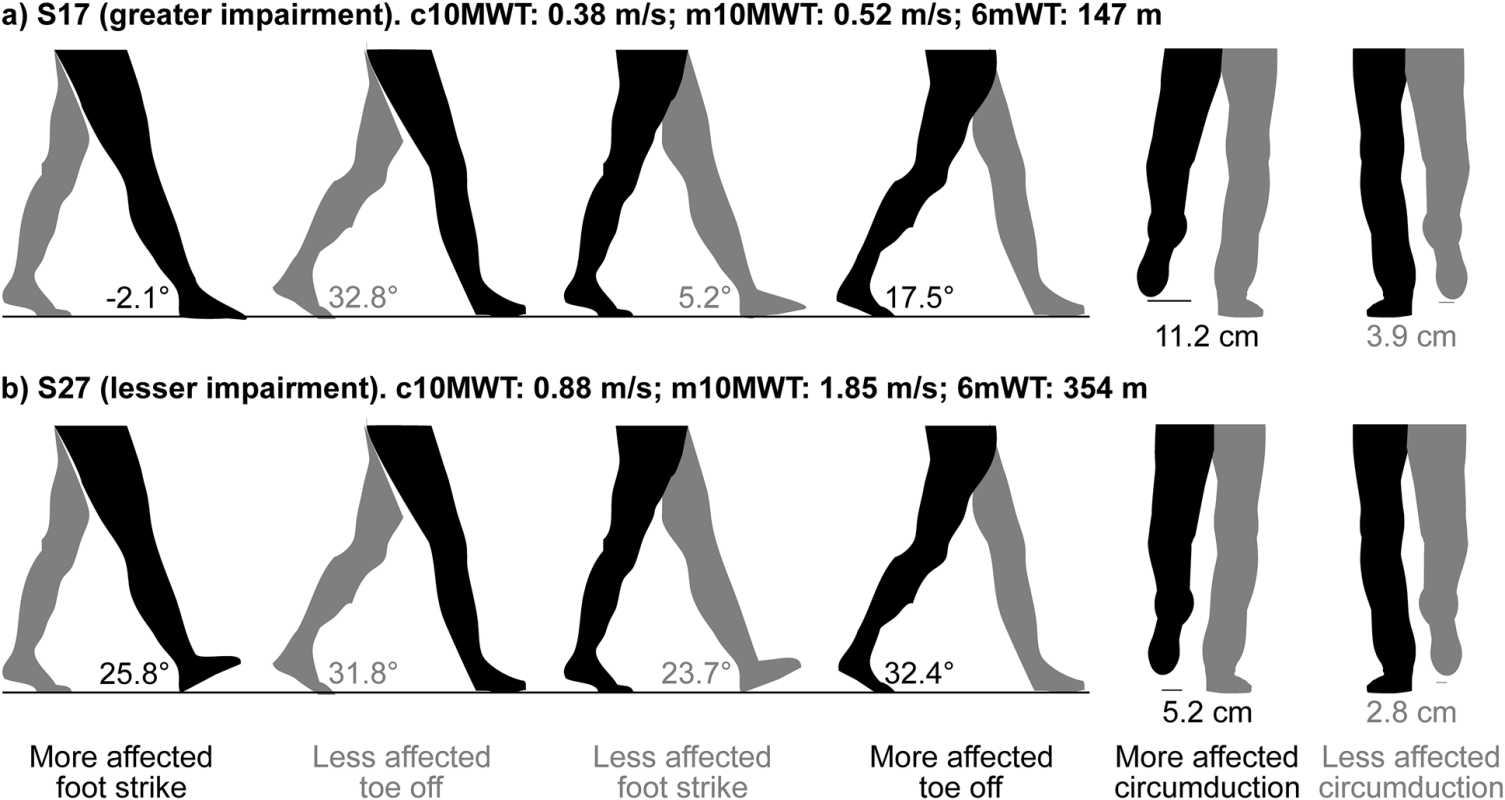
Representative examples of foot strike angle, toe-off angle, and circumduction. The foot strike angle, toe-off angle (°), and circumduction (cm) are shown for the more affected leg (black) and less affected leg (gray) in **a** a participant with greater impairment and **b** a participant with lesser impairment. The participant with greater impairment walked at much slower speeds and had reduced endurance as compared to the individual with lesser impairment. Consistent with the group results, the participant with greater impairment had reduced foot strike angle in the more affected leg (frame 1) and less affected leg (frame 3) and reduced toe-off angle in the more affected leg (frame 4)

Fig. 3 shows correlations of circumduction (a, b), foot strike angle (c, d), and toe-off angle (e, f), in the more (left column) and less affected (right column) limb with 6 mWT distance (endurance). We found significant correlations of greater 6 mWT distance with greater circumduction in the less affected limb (*R* = 0.32, *p* = 0.004), greater foot strike angle in the more affected (*R* = 0.60, *p* < 0.001) and less affected limb (*R* = 0.63, *p* < 0.001), and greater toe-off angle in the more affected (*R* = 0.62, *p* < 0.001) and less affected limb (*R* = 0.74, *p* < 0.001). No walking outcomes were associated with elevation at midswing (*p* > 0.17). Fig. 4 shows representative examples of foot stroke angle, toe-off angle, and circumduction for one participant with greater and lesser impairment. These representative examples show that the participant with greater impairment had smaller toe-off and foot strike angles.

Results for multiple linear regressions are shown in Table 3 for the independent variables of comfortable and maximal walking speed and walking endurance. Across these regressions, we found that better walking function was commonly predicted by greater foot strike angle (more affected and/or less affected) and greater toe-off angle (more affected and/or less affected).

**Table 3:** Multiple linear regressions

Predictors	Unstandard. Beta	Standard. Beta	t	p
IV: c10 mWT				
(Intercept)	− 0.136		− 1.5	0.140
LA foot strike angle	0.011	0.36	3.9	< 0.001
LA toe-off angle	0.016	0.36	4.3	< 0.001
MA toe-off angle	0.010	0.43	4.1	< 0.001
Overall Model	R^2^ = 0.73	Adjusted R^2^ = 0.71	F(3,62) = 54.9	*p* < 0.001
IV: m10 mWT				
(Intercept)	− 0.260		− 1.7	0.090
LA foot strike angle	0.020	0.41	4.2	< 0.001
LA toe-off angle	0.021	0.31	3.6	0.001
MA toe-off angle	0.015	0.27	2.8	0.006
Overall Model	R^2^ = 0.71	Adjusted R^2^ = 0.69	F(3,62) = 50.0	*p* < 0.001
IV: 6 mWT				
(Intercept)	− 23.0		− 0.6	0.560
LA toe-off angle	6.5	0.47	4.1	< 0.001
MA foot strike angle	3.1	0.27	2.6	0.010
MA toe-off angle	2.8	0.25	2.3	0.030
Overall Model	R^2^ = 0.67	Adjusted R^2^ = 0.64	F(3,40) = 26.9	*p* < 0.001

## Discussion

The goal of this project was to measure joint-level kinematic patterns during walking with wearable sensors and determine the association with walking speed and endurance in individuals with chronic stroke. We extracted outcomes from wearable sensors with the intention of representing the hemiparetic gait patterns of stiff-knee gait, foot drop, reduced push-off, hip/pelvic hiking, circumduction, and stepping gait. To highlight the feasibility of these results for clinicians, we used one of the most clinician-friendly sensor systems (APDM Opal), and selected outcome variables that are automatically produced by the analysis software. Across our analyses, we found consistent evidence that greater push-off (toe-off angle) and lesser foot drop (foot strike angle) were the most important predictors of greater walking speed and endurance.

All the predictive models we generated (walking speed and endurance) included the toe-off angle for both the less affected and more affected leg. Specifically, greater toe-off angle (more plantarflexion) in both legs was predictive of faster walking speed and greater walking endurance. These findings demonstrate that individuals with greater push-off (regardless of the leg) had better walking function. Greater toe-off angle is directly associated with greater propulsive force [50], which is important for progressing the center of mass and generating swing phase knee flexion [51, 52]. Hence, it’s clear why greater toe-off angle would contribute to faster walking speeds and greater endurance. Previous work has shown a similar association between the degree of push-off and walking speed [19, 53]. In line with these findings, a number of studies have shown that greater ankle force and power generation at toe-off are associated with faster walking speed [10, 13, 18, 25, 26, 54].

All the predictive models we generated (walking speed and endurance) also included foot strike angle. For walking speed, we found that foot strike angle in the less affected leg was important, while for walking endurance, foot strike angle in the more affected leg was important. Associations were positive, meaning that greater dorsiflexion at foot strike (more heel contact), was associated with faster walking and greater endurance. Said in an alternative way, those with less foot drop had better walking function. The kinematics of the ankle at heel strike are thought to ensure a smooth transition of the center of mass, aiding energy efficiency [55]. Hence, greater plantarflexion at heel strike may impair the smoothness of center of mass transitions and make walking less energy efficient, ultimately resulting in slower walking speeds and decreased endurance. Previous work has had mixed findings about the relation between heel strike angle and walking function. Olney & Richards also found that greater foot strike angle was associated with faster walking speed [19], but Kim & Eng did not find such an association [18]. It is important to note that the sample in the Kim & Eng study had slower walking speeds than in the current study. In a slower sample, foot drop may be more prevalent, there may be a reduced range of foot strike angles or walking speeds, and compensatory strategies may be more beneficial for achieving faster speeds. In longitudinal work, Paoloni et al. found that following segmental muscle vibration in individuals with chronic stroke, there was increase in dorsiflexion of both legs at heel strike, which happened alongside improvements in walking speed [56]. This bolsters the argument that increased dorsiflexion at heel strike may be a contributor to better walking function.

Greater circumduction in both legs was correlated with faster speeds and greater endurance, suggesting that compensation through circumduction may help walking function. Some other studies have found a positive association between greater circumduction and greater walking speed [18, 30], but others have not [29]. Despite the correlation between circumduction and walking function, circumduction was not included in the multiple linear regression models. This suggests that, if possible, better walking function is achieved by avoiding compensatory movements like circumduction. Joint-level kinematical patterns more similar to individuals without stroke may be more optimal. In other words, although circumduction can help achieve better walking function, it is likely inferior to restoring more typical joint-level kinematical patterns. Some research has suggested that circumduction is not actually a compensatory strategy, but rather reflects abnormal coordinative patterns [57, 58].

Elevation at midswing was not correlated with walking speed or endurance. Based on Montane et al., we expected to find that greater elevation would be associated with faster speeds [59]. However, that study specifically targeted limb clearance using three-dimensional gait analysis. There are a couple reasons why we may not have found an impact of elevation at midswing on walking function. First, elevation at midswing reflects the toe elevation at midswing, which is driven by several variables. Elevation at midswing may be lesser with stiff-legged gait but may be greater with compensatory movements like hip hiking, circumduction, and hip flexion. It is hard to parse out individual gait patterns, potentially yielding a mixed result that is not significant.

### Limitations

It’s important to consider that joint-level kinematic patterns are influenced by sex, age, and BMI, among other factors [60]. Also, joint-level kinematic patterns are often inherently linked with the speed of walking, and it is difficult to parse out causation. For example, studies that have varied walking speed within participants with stroke have found that faster walking speeds increases knee flexion, trailing limb angle, and hip hiking, but not circumduction [31, 61]. These speed-dependent effects could have influenced our results, essentially leading to a re-statement that slower walkers walk slower. However, because the direction of causation is unclear (and indeterminable from this study), the relations demonstrated here may still provide important insights.

### Clinical Implications & Future Directions

There are some important implications of this work for the clinic and research. First, joint-level kinematic patterns have traditionally only been accessible via motion capture systems. The current work shows that wearable sensors may be useful for assessing some of these stroke-related and compensatory joint-level kinematic patterns. Notably, wearable sensors are a fraction of the cost, take up minimal space, and are easier to operate than motion capture systems. Wearable sensors have shown good agreement with motion capture systems, including for variables such as heel strike angle, suggesting that they provide a valid assessment of kinematic patterns [33, 35, 62].

In this study, we used joint-level kinematic patterns that are automatically calculated and output from APDM Mobility Lab software that pairs with the APDM Opal sensors. This was done to ensure that the data used in this study could easily be obtained by researchers and clinicians without the technical expertise and computational requirements to analyze the raw sensor output from the wearable sensors. Although we used this particular sensor system, it is likely that the results would generalize to other sensor systems, and possibly to camera or smartphone-based systems (such as those discussed in the Introduction). Other approaches, such as camera systems, may also provide additional benefits such as measuring patient engagement and enhancing virtual reality rehabilitation approaches [63, 64].

Using wearable sensors in individuals with stroke can provide insight into joint-level stroke-related walking deficits and compensatory patterns that could negatively affect walking function. Therapy can then be targeted toward specific deficits or compensatory patterns to improve walking. For example, clinicians could work with patients to improve their toe-off or foot strike angles with the aim of enhancing walking speed and endurance. Changes in these metrics over time could be tracked with a wearable sensor system. Future directions include investigating the longitudinal effects of targeted interventions on joint-level kinematic patterns and exploring the integration of wearable sensor assessments into routine clinical practice.
